# The evaluation of endodontic flare-ups and their relationship to various risk factors

**DOI:** 10.1186/s12903-015-0135-2

**Published:** 2015-11-14

**Authors:** Emel Olga Onay, Mete Ungor, A. Canan Yazici

**Affiliations:** Department of Endodontics, Baskent University, School of Dentistry, 82 sok No 26, 06490 Bahcelievler, Ankara Turkey; Department of Biostatistics, Baskent University, School of Medicine, Ankara, Turkey

**Keywords:** Flare-up, Irrigation, Post-operative pain, Root canal treatment, Visit type

## Abstract

**Background:**

To evaluate the incidence of flare-ups and identify the risk factors including age, gender, tooth type, number of root canals, initial diagnosis, the type of irrigation regimen, treatment modality and the number of visits, in patients who received root canal treatment from January 2002 to January 2008.

**Methods:**

Records of 1819 teeth belonging to 1410 patients treated by 1 endodontics specialist during 6-year period were kept. Patient, tooth, and treatment characteristics were evaluated and the relationships between these characteristics and flare-ups were studied. Statistical analysis was carried out by using Pearson Chi-square test, Fisher’s Exact test, and Binary Logistic regression analyses.

**Results:**

The incidence of flare-ups was 59 (3.2 %) out of 1819 teeth that received endodontic therapy. Pulpal necrosis without periapical pathosis was the most common indication for flare-up (6 %) (*p* < 0.01). Teeth undergoing multiple visits had a higher risk of developing flare-ups compared to those with single appointments (OR: 3.14, CI: 1.414–7.009, *p* < 0.01). There were also no statistically significant differences in the incidence of flare-ups regarding to age, gender, tooth type, number of root canals, treatment modality, and the irrigation solutions that used during the treatment.

**Conclusions:**

The incidence of flare-up is minimal when teeth are treated in one visit. Absence of a periapical lesion in necrotic teeth is a significant risk factor for flare-ups.

## Background

A flare-up following a root canal treatment appointment is a significant problem. The term flare-up is used commonly to describe the development of pain and or swelling which commences a few hours or days after root canal procedures and is of significant severity to require an unscheduled visit for emergency treatment [1]. Lack of exact definition of flare-up resulted in estimated frequency differences from as low as 0.39 % [2] to 20 % [3]. Iqbal et al. [2] stated that in the absence of any gold standard, and because of the variable definitions, comparison of flare-up incidence across studies is challenging.

There are numerous reasons to identify risk factors for flare-ups. An institution may wish to undertake an internal study, identify risk factors, and perhaps change protocols to improve results. If properly identified, the peri-operative predictors of flare-up combined with the clinician’s experience can help to better manage patients post-operatively [2]. Several risk factors have been studied to elucidate which factors could be correlated with the occurrence of flare-ups. These include the number of sessions to complete the treatment [4]; intracanal medication used [5]; host factors, such as gender, age, and dental group [6]; presence of preoperative pain of periapical origin [7]; pulpal diagnosis [8]; periradicular diagnosis [9]; type of treatment, whether initial treatment or retreatment [1]; presence of irritants inside the radicular canal system [7]; apical extrusion of debris; and whether or not apical patency was maintained during preparation [10].

Among these factors, the role of microorganisms and their by-products is well established [1]. Therefore, ideal antimicrobial treatment protocol for teeth with apical periodontitis should be able to eliminate bacteria as well as microbial virulence factors, which might contribute to the perpetuation of periapical inflammation process [11]. A number of antibacterial and chelating substances have been recommended for cleaning and shaping of root canals. Among these substances, sodium hypochlorite (NaOCl) and chlorhexidine (CHX) are two common intracanal irrigants that have demonstrated good antibacterial activity [12]. Harrison et al. [13] found that there was a higher incidence and degree of pain in patients whose canals were either not irrigated or irrigated with saline solution, compared with those irrigated with 5.25 % sodium hypochlorite and 3 % hydrogen peroxide solutions. Despite its good antimicrobial activity, NaOCl has a significant toxicity when extruded into periradicular tissues [14]. Therefore, it is essential to avoid apical extrusion during irrigation to not to contribute to interappointment discomfort.

The aim of this study was to evaluate the incidence of flare-ups and identify the risk factors that may affect them. Patient and treatment factors such as; age, gender, tooth type, number of root canals, initial diagnosis, the type of irrigation regimen, treatment modality and the number of visits were studied for association with the occurence of flare-ups.

## Methods

This study was conducted as a retrospective study at the Department of Endodontics, Baskent University. This study was approved by Baskent University Institutional Review Board and Ethics Committee (Project no: D-KA15/20) and supported by Baskent University Research Fund. The need for an informed consent was waived by Baskent University Institutional Review Board and Ethics Committee listed above because this study used currently existing data collected during the course of routine endodontic treatment and did not pose any additional risks to the patients. Data were gathered on 1819 teeth belonging to 1410 patients visits from January 2002 to January 2008. All of the teeth were treated by the same operator. Each patient’s record consisted of the following data: age, gender (Table 1), tooth type, number of root canals (Table 2), pulpal and periradicular diagnosis of the tooth (Table 3), chemical agents used for irrigation (Table 4), treatment modality (Table 5), number of sessions needed to complete the root canal treatment (Table 6).

**Table 1:** Occurrence of flare-ups according to age group and gender

Prognostic factor		Total no. of teeth	No. of flare-ups	%	*p* value
Age	<20	152	3	2	0.394
	20–29	353	17	4.8	
	30–39	290	11	3.8	
	40–49	336	11	3.3	
	50–59	424	10	2.4	
	>60	264	7	2.7	
Gender	Female	1147	35	3.1	0.584
	Male	672	24	3.6

**Table 2:** Flare-ups in different arches and tooth groups

Tooth group	Total no. of teeth	No. of flare-ups	%	*p* value
Maxillary				0.976
Anterior	298	11	3.7	
Premolar	351	10	2.8	
Molar	395	14	3.5	
Mandibular				
Anterior	100	4	4	
Premolar	236	7	3	
Molar	439	13	3	
No. of root canals				
Single-rooted	743	24	3.2	
Multi-rooted	1076	35	3.3

**Table 3:** Occurence of flare-ups according to pulpal and periradicular diagnosis

Diagnosis	Total no. of teeth	No. of flare-ups	%	*p* value
Normal	43	0	0	0.001
Irreversible pulpitis	1108	24	2.2	
Pulpal necrosis without periapical pathosis	67	4	6	
Pulpal necrosis with periapical pathosis	601	31	5.2

**Table 4:** Occurence of flare-ups according to different irrigation protocols

Irrigation regimen	Total no. of teeth	No. of flare-ups	%	*p* value
NaOCl	1295	36	2.8	0.113
NaOCl + CHX	167	13	7.8	
NaOCl + EDTA + NaOCl	149	3	2	
NaOCl + EDTA + NaOCl + CHX	41	1	2.4	
CHX	150	6	4	
EDTA + CHX	17	0	0

**Table 5:** Occurence of flare-ups according to treatment modality

Treatment modality	Total no. of teeth	No. of flare-ups	%	*p* value
Initial treatment	1680	54	3.2	0.801
Retreatment	139	5	3.6

**Table 6:** Occurence of flare-ups according to no. of appointments

Sessions	Total no. of teeth	No. of flare-ups	%	Odds ratio	95 % CI	*p* value
Single	594	7	1.2	3.14	1.414–7.009	0.001
Multiple	1225	50	4.1			

Root canal treatment necessity for teeth with the initial diagnosis of normal pulp and periradicular status (Table 3) was classified and described as follows [15]:

1. Deep carious lesions: Lesions extending to the pulp chamber without any symptoms of pulpitis; however, requiring root canal treatment due to extensive pulpal exposure. The pulp is vital and there is no periapical radiolucency.
2. Prosthetic purposes: Teeth without any pulpal or periradicular pathosis; however, with the necessity of a prophylactic endodontic intervation due to prosthodontic reasons (e.g. requirement of extensive tissue removal during root canal treatment that will result in pulpal exposure).

While classifying teeth as such, chronic apical periodontitis, acute apical abscesses and chronic apical abscesses were categorized as teeth with periapical pathology, whereas the remaining necrotic cases, which were diagnosed as acute apical periodontitis were regarded as teeth without periapical pathology [15] (Table 3).

Root canal treatment was provided to patients under controlled and standardized conditions. Each tooth was anesthetized with a local anesthetic. A rubber dam was placed, and the operative field was decontaminated with 2.5 % NaOCl. Conventional straight-line access preparations were performed. The initial working length was then determined with an electronic apex locator (Root ZX; J. Morita, Tokyo, Japan). Preflaring was not done before working length determination. Then the working length was established at 0.5 mm up to the radiographic apex by taking a periapical radiographic image. After the middle and coronal third was prepared using ISO size 050, 070 and 090 Gates-Glidden drills (Maillefer, Ballaigues, Switzerland), the root canals were prepared with the step-back technique to an apical size 35–50 depending on the size of the first file that bind at the apical portion of the canals. The preparation was carried out with manually used nickel-titanium files (Maillefer, Ballagigues, Switzerland) under thorough irrigation. During the irrigation protocol 1.25 % NaOCl, 17 % ethylenediaminetetraacetic acid (EDTA), 0.2 % CHX solutions were used in different combinations. To avoid the formation of orange-brown precipitate that contains para-chloraniline, an inter-mediate intracanal flush with distilled water was applied to remove residues of NaOCl, before the use of CHX [16]. The irrigation protocols that used during the treatment were summarized in Table 4.

In retreatment cases, root canal preparations were completed after removal of the previous root canal filling with Gates-Glidden drillls, chloroform and hand files as described above.

In the multiple-visit group, additional sessions were required in the event of an abscess, retreatment, when there was lack of time, when the patient felt tired, or in cases of greater complexity. Under these circumstances, a calcium hydroxide paste (Merck, Darmstadt, Germany) was used to fill the canals, and a temporary seal (Cavit, ESPE, Seefeld/Oberbay, Germany) was placed. Root canal filling was accomplished by using a cold lateral condensation or warm vertical condensation techniques that combined gutta-percha points with AH Plus (Dentsply. De Trey, Konstanz, Germany) sealer using finger spreader.

In the analysis, flare-up was used as a singular outcome variable. Patients categorized to have undergone flare-up when they reported for an unscheduled visit and active treatment suffering from severe pain and/or swelling after initiation or continuation of root canal treatment. Simply reassuring the patient without prescribing medication did not constitute a flare-up. Patients who reported severe pain or swelling but refused an unscheduled visit were not included. Patients who reported pain on normally scheduled second appointment were not categorized as flare-ups.

Statistical analysis was carried out by using Pearson Chi-square test, Fisher’s Exact test, and Binary Logistic regression analyses.

## Results

The overall incidence of flare-ups was 59 (3.2 %) out of 1819 teeth that received endodontic therapy. There were no statistically significant differences in the incidence of flare-ups regarding the following factors: age and gender (Table 1); tooth type and the number of root canals (Table 2); chemical agents used for irrigation (Table 4) (*p* > 0.05). There was also no difference regarding the occurrence of flare-up between the initial treatment group (3.2 %) and retreatment group (3.6 %) (*p* > 0.05). Data are summarized in Table 5. Pulpal necrosis without periapical pathosis was the most common indication for flare-ups (6 %) followed by pulpal necrosis with periapical pathosis (5.2 %) and irreversible pulpitis (2.2 %) (*p* < 0.01; Table 3). Teeth undergoing multiple visits had a higher risk of developing flare-ups compared to those with single appointments (OR: 3.14, CI: 1.414–7.009, *p* < 0.01; Table 6).

## Discussion

For many patients flare-up is an unpleasant experience, which brings skepticism about their dentist skills. Hargreaves et al. [17] indicated that every clinician who provides root canal treatment had to deal with this misconception and the clinician’s skill is often primarily judged by the success or failure of pain control. Despite judicious and careful treatment procedures, complications such as pain, swelling or both may occur. As with emergencies occurring prior to root canal treatment, these interappointment emergencies are undesirable and disruptive events and should be resolved immediately. Occasionally flare-ups are unexpected, although they can often be predicted according to certain patient presenting factors [18].

Analysis regarding the influence of a patient age, gender, the tooth and arch under consideration, as well as of the number of root canals, did not show statistically significant differences in the flare-up rates. These results corroborate the findings of other authors [8, 9, 19, 20]. Conversely, a retrospective study carried out by Torabinejad et al. [6] showed a positive correlation between flare-up rates and age, gender, and jaw location.

In the present study, the relationship between the initial diagnosis and flare-up was evaluated and the absence of periapical lesion in necrotic teeth found to be a significant predictor of flare-up. Our results were consistent with the study carried out by Torabinejad et al. [6] who linked the reason with the inadequate space available for the dispersal of the pressure due to acute periradicular inflammation. On the other hand, Iqbal et al. [2], de Olivera Alves [20], and Tanalp et al. [15] indicated cases with a periapical lesion had a higher risk of developing pain and flare-ups compared to those with no periapical involvement. However, other researchers [21, 22] were not able to find a relationship between radiolucency and acute exacerbation. The reason for the difference cannot be readily explained but could relate to different patient population, which only consists of necrotic teeth, varying treatment modalities, and other methods of assessment.

The present study have indicated the type of irrigation solution used makes no difference in the incidence of postoperative discomfort, which also corroborates the findings of other authors [23, 24] who showed that neither the individual use nor the combined use of irrigation solutions are associated with increased interappointment pain. The contribution of antimicrobial treatment protocol to the incidence of flare-up remains controversial. The induction of pain in root canal treatment is multifactorial, it is difficult to attribute a lower pain incidence specifically to the use of any particular irrigation solution.

Many investigations have conducted studies on the antibacterial effectiveness of CHX in different concentrations. It has been demonstrated that the antibacterial efficacy and substantivity of CHX evidently depends on its concentration level [25, 26]. On the other hand, because high concentrations of irrigants cannot be ubiquitously delivered to all sites in the root canal due to dilution and the complexity of the root canal system, Ma et al. [27] tested low concentration (0.2 %) of CHX versus 2 % CHX. They stated that the 2 and 0.2 % CHX treatments significantly decreased the plaktonic and biofilm Enterococcus faecalis survival rates in the alkaline conditions. In the present study 0.2 % CHX was used as an irrigation solution. Further investigations are necessary to compare the effectiveness of 2 % CHX on the incidence of flare-ups compared to other protocols in patients with similar pulpal and periradicular conditions.

Analysis of the type of treatment performed whether initial treatment or retreatment showed no statistically significant difference regarding the incidence of flare-ups. This was consistent with the study carried out by Iqbal et al. [2], Siqueira et al. [7], and de Oliveira Alves [20]. Interestingly, Trope [28] found an 8-fold higher (13.6 %) incidence of flare-ups in retreatment cases involving teeth with periapical periodontitis treated in single appointments. This might be a result of the sample type, inclusion and exclusion criteria, and standardization of clinical factors controlled by operators or those evaluated by the patients.

Single- versus multiple-visit root canal treatment has been the subject of long-standing debate in the endodontic community [29]. The statistically significant occurrence of more flare-ups in the multiple-visit group than in the single-visit group in the present study agrees with the reports of other authors [8, 15]. The lower incidence of pain in the single-visit group may be attributed to immediate obturation, which eliminates bacterial ingress from a leaky restoration [30]. Still, another possible reason may be the greater tendency of treating vital and nonproblematic cases in one visit [8]. Conversely, there is a common belief that multiple visits with interappointment medicament application could minimize the incidence of flare-ups in teeth with periapical pathology and a necrotic pulp [31]. A positive relationship between single visit and flare-up has been previously reported [5, 32]. On the other hand, a majority of authors comparing these two approaches did not find any difference regarding the incidence of flare-ups [2, 9, 19, 20, 33].

The retrospective design of the present study might be considered as a limitation, which lacks the ability to established temporal relationships. Despite this drawback, retrospective studies are less prone to be biased by the investigators’ opinions than prospective studies. Furthermore, random selection of cases and a large sample size, both of which are critical to clinical studies involving many variables, are generally easier to achieve in a retrospective study than in a prospective study [6]. However, the study design with greatest power is the randomized-controlled trial because it can minimize confounders, maximize control over environment, and providing the most convincing casual relationship [34]. Future randomized-controlled trials with well-defined inclusion criteria are needed to fully define all of the factors contributing to flare-ups associated with root canal treatment.

## Conclusions

Within the parameters of this study, it can be concluded that the incidence of flare-up is minimal when teeth are treated in one visit. Absence of a periapical lesion in necrotic teeth is a significant risk factor for flare-ups.

## Abbreviations

NaOCl: Sodium hypochlorite
EDTA: Ethylenediaminetetraacetic acid
CHX: Chlorhexidine gluconate
